# Participation in breast cancer screening among women of Turkish origin in Germany – a register-based study

**DOI:** 10.1186/1472-6874-14-24

**Published:** 2014-02-10

**Authors:** Eva-Maria Berens, Lisa Stahl, Yüce Yilmaz-Aslan, Odile Sauzet, Jacob Spallek, Oliver Razum

**Affiliations:** 1 Department of Epidemiology and International Public Health, Bielefeld University, School of Public Health, Universitaetsstrasse 25, 33615 Bielefeld, Germany

**Keywords:** Breast cancer screening, Mammography, Participation, Use, Uptake, Attendance, Immigrants, Turks, Germany, Name-based identification

## Abstract

**Background:**

Population-based breast cancer screening programs were implemented to reduce breast cancer mortality and to improve recovery chances. Breast cancer screening participation among migrant women differs from that of autochthonous populations in several European countries. Here we investigate for the first time participation among women of Turkish origin in Germany.

**Methods:**

Data of five screening units covering 2010 and 2011 as well as associated population registries were analysed. Women of Turkish origin were identified using a name-based algorithm. Participation ratios among women of Turkish origin and odds ratios compared to women of non-Turkish origin were calculated. Analyses were stratified and adjusted for age-groups and screening unit.

**Results:**

A total of 208,500 participants in the five breast screening units were included, out of 423,649 eligible women in the catchment areas (participation 49.2%). Women of Turkish origin have a slightly higher chance to participate in breast cancer screening than women without Turkish origin (OR 1.17; 95% CI: 1.14-1.21). Only women of Turkish origin aged 65–69 years have a lower chance to participate than women without Turkish origin (OR: 0.71; 95% CI: 0.66-0.75).

**Conclusion:**

In spite of low participation in preventive measures among migrant populations, the overall breast cancer screening participation among women of Turkish origin in Germany seems to be higher compared to women of non-Turkish origin. Turkish women aged 65 years and above have a lower chance of participation than younger Turkish women. There is need for further research to study factors affecting participation in screening among migrant and non-migrant populations in Germany.

## Background

Breast cancer is the most common cancer among women world-wide [1]. In Europe, there were 331,000 breast cancer cases in 2006, accounting for 30 percent of all cancer cases among women; with approximately 90,000 deaths in 2006, breast cancer is the second most common cause of death among women in Europe [2]. Based on European guidelines, population-based breast cancer screening programs were implemented in most countries to reduce breast cancer mortality and start treatment earlier and thus to enhance recovery chances [3].

In Germany, a nationwide population-based breast cancer screening program was implemented from 2005 onwards [4]. Implementation has been completed in 2009 and about 10 million women between the ages of 50 and 69 years are served by 94 screening units. Women in the eligible age group are invited by mail to undergo a mammography in the specialised screening unit in their area of residence every other year [5]. By 2009, more than half (54.4 percent) of the women officially invited actually participated in the breast cancer screening program (this figure includes some who attended before the automatically generated invitation reached them). The European guidelines recommend a participation ratio of at least 70 percent to ensure effectiveness of the screening program [3]. In 2005–2006, a slightly larger proportion of women participated (57.3 percent) than in 2007 (53.6 percent) [5]. There are large regional differences in screening participation: In the federal states of Bavaria and Schleswig-Holstein, 45.4 percent of the eligible women attended the screening in 2009 while in Saxony, Bremen, Saxony-Anhalt and Mecklenburg-West Pomerania, the ratio was over 60 percent [5].

Factors affecting the probability of failing to participate in screening have been shown to include older age, lower educational status, not owning a car, having no children and no partner and several other factors [6-10]. In addition, there is evidence for differences in breast cancer screening participation between migrant and non-migrant women in several European countries [8,9,11-13]. For example, Lagerlund et al. [6] show a lifetime participation of 90 percent among Swedish women, while participation among migrant women from non-Scandinavian European countries, South America and Asia (80 percent), Africa (76 percent) and North America (73 percent) was lower. A study from the Netherlands shows a participation of 44 percent among Turkish migrants (1995–2005) compared to an overall participation of 79 percent. Older Turkish women were less likely to participate (40 percent) than younger Turkish women (50 percent) [14]. Participation among Turkish migrants increased from 50 percent in 1997–1998 to 62 percent in 2007–2008. This, however, is still much lower than the overall participation of 80 percent in 1997–1998 and 83 percent in 2007–2008 [15].

Germany hosts a total of 16 million migrants (for the purpose of this paper defined as immigrants or their direct descendants), which is equivalent to more than 19 percent of its population. About 3 million people (more than 3 percent of the total population and almost 20 percent of the migrant population) residing in Germany are of Turkish origin. Among the group of Turkish migrants there are about 334,000 women aged 45 to 75 years [16]. In the next decades the proportion of older (Turkish) migrants will increase considerably [17], and so will the number and the proportion of migrants eligible for breast cancer screening.

There is evidence for a lower participation in preventive measures among migrants in Germany [18]. For example, the participation of adult migrants in routine health examinations is lower than in the majority population [19]. To date there are no studies on breast cancer screening participation among migrants in Germany. Our research questions are: Does the chance to participate in breast cancer screening differ between women of Turkish origin compared to women of non-Turkish origin? And, is the chance to participate in breast cancer screening among women of Turkish origin dependent on their age?

## Methods

### Data sources

The study is based on routine data from screening units and population registries. All women participating in the breast cancer screening program are registered in the datasets of the screening units responsible for a particular geographical region (numerator population). These screening data contain names of participants as well as year of birth. As in Germany there is no nationwide database comprising data on participation and migration status, five screening units from different regions were chosen (Duisburg, Bielefeld, Paderborn, Hamburg, and Berlin). We selected “typical” urban screening units to cover different regions with different proportions of migrants in the population.

To assure data privacy the five screening units were labeled as screening unit A-E, not following the order above. In Germany, women between 50 and 69 years of age are eligible to attend breast cancer screening every two years. Data from one 2-year invitation period (2010 and 2011) were included in the study with the aim to cover all women eligible in this period. Women born in 1940 or 1961 were excluded from the calculation of screening participation as they lost or gained eligibility to screening during the 2-year period. For the presentation of data the age of the women was set as the age in the first study year (2010). Thus, women born in 1960 were labeled as 50 years old and women born in 1941 were labeled as 69 years old.

To calculate participation ratios, the number of women living in the catchment areas of each screening unit was obtained from the population registries of the 50 cities and villages comprising the catchment areas of the five screening units (denominator). One village authority (associated to screening unit E) refused to provide population data for the project and was therefore also excluded in the screening dataset. Population data on January 1^st^ 2011 (midterm of the period) was used.

### Identification of women of Turkish origin

Women of Turkish origin were identified in both datasets by using a name-based algorithm. As Turkish family names were introduced by law in the 1930s and had to have a meaning in the Turkish language, they are thus highly specific. This method allows identifying women of Turkish origin in datasets without any other migration-related variables such as place of birth or nationality [20-22]. Women with Turkish and non-Turkish first and family names are identified by automatically comparing the names to extensive name lists. Ambiguous cases are flagged and checked manually by a Turkish-speaking researcher and then labeled as Turkish or non-Turkish. The methodology of the name-based identification has been described in more detail in previous publications [20,21].

### Analysis

The number of women with and without Turkish origin in each of the screening units was divided by the numbers of women with and without Turkish origin in the respective catchment populations. We calculated odds ratios to compare the chances of participation among women of Turkish origin and among women of non-Turkish origin. At the end of our 2-year study period, all women had a chance to participate in screening. The date of participation within the study period is not of relevance for our purposes; we are interested in comparing the chances of participation within a 2-year screening round. We then compared chances of overall participation adjusted for age-group and screening unit, as well as chances of participation for 5-year age-groups adjusted for screening unit, between women of Turkish origin and women without Turkish origin. The analyses were run using Stata 12.0. The study was approved by the ethical committee of Muenster University.

## Results

A total of 208,500 participants in breast cancer screening were included in the analysis. The automated part of the name-based algorithm identified 5,978 women with Turkish names and an additional 5,086 women with ambiguous names. After the manual check a total of 9,754 participants (4.7 percent of the total participants) were classified as of Turkish origin and 198,746 as of non-Turkish origin. Furthermore, 423,649 women in the eligible age-group living in the catchment areas of the screening units were included. Of these, 11,143 women were identified as having Turkish names and 9,839 women as having ambiguous names. After the manual check a total of 18,658 women (4.4 percent of the total population) in the eligible age group were classified as of Turkish origin and 404,991 as of non-Turkish origin.

Table 1 shows participation ratios and odds ratios stratified by screening unit and 5-year age-groups. About half of the women in the study areas participated in the breast cancer screening program. Participation ratios vary by screening unit from 42.6 percent in screening unit A to 57.5 percent in screening unit D. The chance to participate among all women adjusted for age was 1.81 times higher in screening unit B (95% CI: 1.78-1.85) than in screening unit A (ref). The analysis by 5-year age-groups shows that the overall chance to participate in older women aged 65–69 years was marginally lower (46.7 percent; OR 0.91; 95% CI: 0.89-0.92) than in women in the other age groups where it was about 50 percent. There was no change in overall participation ratios when controlling for migration (data not shown). All regional and age differences in chance of participation are statistically significant (Table 1).

**Table 1:** Number of women in the study and participation in breast screening among all women, 2010/2011

	**Number of women in study (% women of Turkish origin in this group)**	**Overall participation**	
		**[%]**	**OR (95% CI)**^**a**^
**Total**	423,649 (4.4)	49.2	
Screening unit			
A	110,845 (6.0)	42.6	Ref
B	67,565 (7.9)	52.0	1.81 (1.78–1.85)*
C	107,707 (3.8)	46.0	1.45 (1.42–1.48)*
D	82,787 (3.8)	57.5	1.14 (1.12–1.17)*
E	98,632 (1.2)	50.8	1.38 (1.36–1.41)*
Age group (years)			
50-54	125,006 (3.6)	49.6	Ref
55-59	109,586 (4.0)	50.1	1.02 (1.01–1.04)**
60-64	94,852 (5.8)	50.1	1.03 (1.01–1.05)**
65-69	94,205 (4.5)	46.7	0.91 (0.89–0.92)**

^a^An OR >1 means that the chance to participate in breast cancer screening is higher among all women in the respective category than in the reference category.

*adjusted for age-groups.

**adjusted for screening unit.

The participation ratio was slightly higher in women of Turkish origin (52.3 percent) than in women of non-Turkish origin (49.1 percent). The chance in women of Turkish origin to participate in breast cancer screening adjusted for age and screening unit was 17 percent higher compared to women of non-Turkish origin (OR: 1.17; 95% CI: 1.14-1.21) (Table 2).

**Table 2:** Participation in breast screening among women of Turkish origin compared to women of non-Turkish origin, 2010/2011

	**Participation in women of Turkish origin**		**OR (95% CI)**^**a**^
	**n**	**[%]**	
Migration (yes vs. no)	9,754	52.3	1.17 (1.14-1.21)*
Age group (years)			
50-54	2,658	58.9	1.50 (1.42–1.60)**
55-59	2,564	57.9	1.45 (1.36–1.54)**
60-64	2,844	51.5	1.12 (1.06–1.18)**
65-69	1,688	40.2	0.71 (0.66–0.75)**

^a^An OR >1 means that the chance to participate in breast cancer screening is higher among women of Turkish origin than among women without Turkish origin.

*adjusted for age-group and screening unit.

**adjusted for screening unit.

Women of Turkish origin, however, showed declining participation with increasing age (Table 2). This trend cannot be shown for women of non-Turkish origin. A higher proportion (47.0 percent) of women without Turkish origin aged 65–69 years participated in breast cancer screening than women of Turkish origin of the same age-group (40.2 percent). Consequently, the chance for women of Turkish origin aged 65–69 years to attend screening was lower than for women of non-Turkish origin (OR: 0.71; 95% CI 0.66-0.75). In the other age groups the chance to participate was higher among women of Turkish origin than in women without Turkish origin (Table 2). The chance to participate was 50 percent higher in women aged 50–54 of Turkish origin compared to women without Turkish origin adjusted for screening unit. The associations in all age-groups were statistically significant.

## Discussion

### Findings

For the first time, breast cancer screening participation among women of Turkish origin in Germany was analysed. Furthermore, our study presents for the first time data on screening participation in 5-year age-groups based on routine data. The routine evaluation report of breast cancer screening in Germany [4] only contains overall participation ratios. Other studies had to rely on self-reported screening participation in smaller samples e.g. [23,24].

Overall, the participation in breast cancer screening among women of Turkish origin and women of non-Turkish origin is quite similar. Our study shows that women of Turkish origin have a slightly higher chance to participate in breast cancer screening than women of non-Turkish origin. This finding is unexpected when taking into consideration that other studies show a lower participation in preventive measures among migrants in Germany, e.g. a lower participation of adult migrants in routine health screening tests [19]. Our results seem also not to be consistent with European studies showing a lower participation in breast cancer screening among various migrant groups, compared to the autochthonous populations [6,11,13]. However, these studies did not stratify for country of origin, so they fail to take into account that migrant populations are heterogeneous.

A reason for the comparatively small differences in participation ratios among women of Turkish origin and women of non-Turkish origin in Germany could be that participation in breast cancer screening is lower in Germany than in some other European countries. For example, the participation ratio of Turkish immigrant women in the Netherlands (44–62 percent) [14,15] is approximately as high as in Germany (40–59 percent). However, participation among Dutch women is much higher (79–83 percent) than among all women in Germany (43–58 percent), thus leading to a larger differential.

Kristiansen and colleagues [13] have shown a decline in participation among both migrants and the autochthonous Danish population with increasing level of education; this decline was higher among migrant women than among Danish women. There is evidence for differences in educational level among migrants compared to the autochthonous population in Germany. Migrant women aged 45–65 years more often have no formal qualification than women of the same age group in the autochthonous population (more than 15 percent vs. about 1 percent) [16]. In our study no data on socio-economic status was available. However, similar studies from the Netherlands also could not adjust for socio-economic status; they found lower participation ratios among women born in Turkey compared to Dutch women [14,15].

For women of Turkish origin participation ratio was higher the younger they were. This is in line with findings from Visser and colleagues from the Netherlands [14]. Turkish immigrant women aged 50 to 54 years showed a higher participation ratio than older Turkish women. There was no such tendency among Dutch women, which is in line with our findings. A possible explanation could be that older women of Turkish origin more often face problems with the language of the host country than younger ones. Another explanation could be differences in educational levels among women of Turkish origin in different age groups.

Finally, our results confirm a regional variation in screening participation which is in line with regional variations in screening participation reported in other studies in Germany [5] and in the Netherlands [15].

### Methodological strengths and limitations

Our study is based on routine data of breast cancer screening units and thus has a large sample size. Data quality is good as women attending breast cancer screening are registered based on name and date of birth stored on their health insurance card. Administrative procedures require that all women attending the screening are registered in the screening datasets. Thus, registration of participation is complete. Research has shown that self-reported screening participation is often imprecise, especially among migrant populations [25,26]. Our data, however, do not contain additional information on factors which might influence screening participation such as socio-economic status. Thus, our analyses are limited as we could not control for possible confounders of screening participation.

Our approach leads to somewhat lower estimates of overall screening participation (49 percent) than the official evaluation where a participation of 54 percent was reported for 2009 [5]. Albert and colleagues [23] found an even higher participation ratio of 66 percent for Germany in their (albeit much smaller) sample relying on self-reported participation. However there is evidence for overestimation when participation in breast cancer screening is self-reported [26]. One possible explanation for our lower participation ratios lies in the regional variability we observed and the fact that the five screening units we selected may not be representative of Germany as a whole. Other studies have shown that participation in breast cancer screening varies greatly by region. In the federal states of Hamburg a participation of 48.1 percent was reported in 2009, while in Berlin (50.7 percent) and North Rhine-Westfalia (54.3 percent) participation was higher. Variation in participation is even higher at the level of screening units, ranging from 35.5 percent to 71.4 percent in 2009 [5]. Participation in our study ranges from 42.6 percent to 57.5 percent. In addition, migration to a particular place is not random. In General, migrants tend to settle in urban areas in former West Germany rather than in rural areas and former East Germany [27]. Turkish migrant women in Paderborn might be different from those in Berlin. We tried to take this possible variability into account by selecting screening units from different urban regions with different proportions of Turkish migrant women in the population.

A second explanation for our lower estimates of screening participation could be differences in the denominator population. In the official evaluation report screening participation is calculated based on the number of women invited. In Germany, 91.6 percent of the eligible women were invited in 2009 [5]. Using the number of women invited as the denominator population was not possible in our study. We had to base our analyses on the best estimate of the total number of women (invited and not invited) aged 50–69 years old in the catchment areas of the screening units.

We chose January 1^st^, 2011, the middle of the study period, as reference date for the population data. The eligible population is not known exactly due to relocations and death during the study period. We make the implicit assumption that relocation behavior does not differ substantially between Turkish and non-Turkish women aged 50 to 69 years. While it is known that many older Turkish immigrants stay in Turkey for several weeks a year, they keep their residency in Germany during that time [28] and with that their eligibility for breast cancer screening.

The name-based identification of persons of Turkish origin is a good method for migrant-sensitive analyses when no information on migration status is available. The algorithm used in this study has reached a sensitivity of 85 percent in a previous study with cancer registry data. The algorithm discriminated well against Greek and Arab names (1 percent false positive results) [21]. We could not assess the performance of the algorithm in our dataset as additional migration variables were missing. But assessing migration relying on name-based identification has several limitations. Firstly, a name-based algorithm can never identify all persons of Turkish origin in a dataset. For example, women who married a person with a non-Turkish name will probably not be identified. However, the proportion of married Turkish migrant women who married a native German is only 2 percent; 65 percent married a Turkish migrant; and 34 percent are already married at the time of immigration [29]. Given the small proportion of mixed marriages this should not bias our findings. Secondly, we compare Turkish migrant women with the general female population of the respective age (excluding, of course, Turkish migrant women). Our methodology does not allow comparing Turkish migrant women with autochthonous women alone. This limitation is unlikely to bias our findings in a substantial way: There are more than 16 million women aged 45–75 years residing in Germany, and the number of other migrant women among them is not very large. For example, the second-largest subgroup, after Turkish migrant women, are 270,000 women from Poland [16]. Thus, while “other” migrant women are part of our comparison group, they constitute only a small proportion and have limited effect on our estimates.

## Conclusions

The overall participation ratio in breast cancer screening of over 70 percent recommended by EU [3] is not yet reached in Germany. However, our results indicate that, in contrast to other preventive measures, women of Turkish origin participate to a higher proportion in breast cancer screening than women without Turkish origin. But their participation declines with increasing age.

Further studies are needed to verify our results as there are several methodological limitations. Future research should investigate reasons for the possibly higher screening participation among women of Turkish origin; the findings might also inform preventive programs in which participation by migrants is low. Factors affecting participation among migrant and non-migrant populations in different age groups need to be studied to explain possible differences or similarities. Finally, there is a need for studies focussing on reasons for the low overall participation in screening in Germany.

## Competing interests

The authors declare that they have no competing interests.

## Author’s contributions

EMB, JS and OR conceived the idea for the study. EMB, JS, LS and YYA obtained the data. EMB, LS and OS cleaned the data and completed the statistical analysis. EMB wrote the manuscript which was revised by all authors. All authors have read and approved the final manuscript.

## Pre-publication history

The pre-publication history for this paper can be accessed here:

http://www.biomedcentral.com/1472-6874/14/24/prepub

## References

[B1] Boyle P Levin B World Cancer Report 2008 2008 Lyon: International Agency for Research on Cancer

[B2] European Communities Cancer screening in the European Union. Report on the implementation of the council recommendation on cancer screening [http://screening.iarc.fr/doc/cancer_screening.pdf]

[B3] European Communities European guidelines for quality assurance in breast cancer screening and diagnosis [http://screening.iarc.fr/doc/ND7306954ENC_002.pdf]

[B4] Kooperationsgemeinschaft Mammographie Evaluationsbericht 2005–2007 – Ergebnisse des Mammographie-Screening-Programms in Deutschland [http://www.mammo-programm.de/cms_upload/datenpool/evaluationsbericht05-07_web.pdf]

[B5] Kooperationsgemeinschaft Mammographie Evaluationsbericht 2008–2009 – Ergebnisse des Mammographie-Screening-Programms in Deutschland [http://www.mammo-programm.de/cms_upload/datenpool/evaluationsbericht_2008-2009_web.pdf]

[B6] Lagerlund M Maxwell AE Bastani R Thurfjell E Ekbom A Lambe M Sociodemographic predictors of non-attendance at invitational mammography screening–a population-based register study (Sweden) Cancer Causes Control 2002 13 73 82 10.1023/A:1013978421073 11899121

[B7] Puddu M Demarest S Tafforeau J Does a national screening programme reduce socioeconomic inequalities in mammography use? Int J Public Health 2009 54 61 68 19247577 10.1007/s00038-009-8105-6

[B8] Bulliard JL de Landtsheer JP Levi F Profile of women not attending in the swiss mammography screening pilot Breast 2004 13 284 289 10.1016/j.breast.2004.03.001 15325662

[B9] Fontana M Bischoff A Uptake of breast cancer screening measures among immigrant and Swiss women in Switzerland Swiss Med Wkly 2008 138 752 758 19130329 10.4414/smw.2008.12328

[B10] Moser K Patnick J Beral V Inequalities in reported use of breast and cervical screening in Great Britain: analysis of cross sectional survey data BMJ 2009 338 b2025 10.1136/bmj.b2025 19531549 PMC2697310

[B11] Zackrisson S Lindstrom M Moghaddassi M Andersson I Janzon L Social predictors of non-attendance in an urban mammographic screening programme Scand J Public Health 2007 35 548 554 10.1080/14034940701291716 17852976

[B12] Renshaw C Jack RH Dixon S Moller H Davies EA Estimating attendance for breast cancer screening in ethnic groups in London BMC Public Health 2010 10 157 10.1186/1471-2458-10-157 20334699 PMC2850886

[B13] Kristiansen M Thorsted BL Krasnik A Euler-Chelpin von M Participation in mammography screening among migrants and non-migrants in Denmark Acta Oncol 2012 51 28 36 10.3109/0284186X.2011.626447 22035117

[B14] Visser O Am van P Ory FG van Leeuwen FE Results of breast cancer screening in first generation migrants in Northwest Eur J Cancer Prev 2005 14 251 255 10.1097/00008469-200506000-00009 15901994

[B15] Vermeer B Van den Muijsenbergh ME The attendance of migrant women at the national breast cancer screening in the Netherlands 1997–2008 Eur J Cancer Prev 2010 19 195 198 10.1097/CEJ.0b013e328337214c 20150815

[B16] Statistisches Bundesamt Bevölkerung mit Migrationshintergrund - Ergebnisse des Mikrozensus 2011 - Fachserie 1 Reihe 2.2 [https://www.destatis.de/DE/Publikationen/Thematisch/Bevoelkerung/MigrationIntegration/Migrationshintergrund2010220117004.pdf?__blob=publicationFile]

[B17] Ströker K Modellierung von Szenarien zur zukünftigen Entwicklung der Bevölkerung mit Migrationshintergrund in NRW [http://www.it.nrw.de/statistik/analysen/stat_studien/2007/band_42/stroeker_42.pdf]

[B18] Spallek J Zeeb H Razum O Prevention among immigrants: the example of Germany BMC Public Health 2010 10 92 10.1186/1471-2458-10-92 20181232 PMC2837628

[B19] Zeeb H Baune BT Vollmer W Cremer D Krämer A Gesundheitliche Lage und Gesundheitsversorgung von erwachsenen Migranten - ein Survey bei der Schuleingangsuntersuchung Gesundheitsw 2004 66 76 84 10.1055/s-2004-812825 14994205

[B20] Razum O Zeeb H Beck K Becher H Ziegler H Stegmaier C Combining a name algorithm with a capture-recapture method to retrieve cases of Turkish descent from a German population-based cancer registry Eur J Cancer 2000 36 2380 2384 10.1016/S0959-8049(00)00333-6 11094313

[B21] Razum O Zeeb H Akgun S How useful is a name-based algorithm in health research among Turkish migrants in Germany? Trop Med Int Health 2001 6 654 661 10.1046/j.1365-3156.2001.00760.x 11555431

[B22] Spallek J Kaatsch P Spix C Ulusoy N Zeeb H Razum O Name-based identification of cases of Turkish origin in the childhood cancer registry in Mainz Gesundheitsw 2006 68 643 649 10.1055/s-2006-927166 17099826

[B23] Albert US Kalder M Schulte H Klusendick M Diener J Schulz-Zehden B Kopp I Nass-Griegoleit I The population-based mammography screening programme in Germany: uptake and first experiences of women in 10 federal states Gesundheitsw 2012 74 61 70 10.1055/s-0030-1268441 21229475

[B24] Nass-Griegoleit I Schultz-Zehden B Klusendick M Diener J Schulte H Studie belegt hohe Akezptanz des Mammographie-Screenings bei Frauen Frauenarzt 2009 50 494 501

[B25] Baron-Epel O Friedman N Lernau O Validity of self-reported mammography in a multicultural population in Israel Prev Med 2008 46 489 491 10.1016/j.ypmed.2008.03.003 18407343

[B26] Cronin KA Miglioretti DL Krapcho M Yu B Geller BM Carney PA Onega T Feuer EJ Breen N Ballard-Barbash R Bias associated with self-report of prior screening mammography Cancer Epidemiol Biomarkers Prev 2009 18 1699 1705 10.1158/1055-9965.EPI-09-0020 19505902 PMC2771779

[B27] Rühl S Grunddaten der Zuwandererbevölkerung in Deutschland 2009 Nürnberg: Bundesamt für Migration und Flüchtlinge [Bundesamt für Migration und Flüchtlinge, Referat 220 (Series Editor): *Integrationsreport*, vol.6]

[B28] Bundesministerium für Familie SFuJ Fünfter Bericht zur Lage der älteren Generation in der Bundesrepublik Deutschland. Potenziale des Alters in Wirtschaft und Gesellschaft. Der Beitrag älterer Menschen zum Zusammenhalt der Generationen. Bericht der Sachverständigenkommission an das Bundesministerium für Familie, Senioren, Frauen und Jugend [http://www.bmfsfj.de/RedaktionBMFSFJ/Abteilung3/Pdf-Anlagen/fuenfter-altenbericht,property=pdf,bereich=,rwb=true.pdf]

[B29] González-Ferrer A Who Do immigrants marry? Partner choice among single immigrants in Germany Eur Sociol Rev 2006 22 171 185

